# Effects of Polypropylene Orientation on Mechanical and Heat Seal Properties of Polymer-Aluminum-Polymer Composite Films for Pouch Lithium-Ion Batteries

**DOI:** 10.3390/ma11010144

**Published:** 2018-01-16

**Authors:** Fangxinyu Zeng, Jinyao Chen, Feng Yang, Jian Kang, Ya Cao, Ming Xiang

**Affiliations:** State Key Laboratory of Polymer Materials Engineering, Polymer Research Institute of Sichuan University, Chengdu 610065, China; nsno1@163.com (F.Z.); chenjinyao@scu.edu.cn (J.C.); yangfeng@scu.edu.cn (F.Y.); caoya@scu.edu.cn (Y.C.); xiangming@scu.edu.cn (M.X.)

**Keywords:** composite film, lamination, heat seal behavior, tensile properties

## Abstract

In this study, polyamide-aluminum foil-polypropylene (PA-Al-PP) composite films with different orientation status of the PP layer were prepared, and their morphology, tensile, peeling and heat seal behavior were studied. The comparative study of tensile and fracture behaviors of single-layer film of PA, Al and PP, as well as the composite films of PA-Al, PP-Al and PA-Al-PP revealed that in PA-Al-PP composite film, the PA layer with the highest tensile strength can share the tensile stress from the Al layer during stretching, while the PP layer with the lowest tensile strength can prevent further development of the small cracks on boundary of the Al layer during stretching. Moreover, the study of heat seal behavior suggested that both the orientation status and the heat seal conditions were important factors in determining the heat seal strength (*HSS*) and failure behavior of the sample. Four failure types were observed, and a clear correspondence between *HSS* and failure types was found. The results also elucidated that for the composite film, only in the cases where the tensile stress was efficiently released by each layer during *HSS* measurement could the composite film exhibit desired high *HSS* that was even higher than its tensile strength.

## 1. Introduction

Compared to other commonly used rechargeable batteries, the lithium ion batteries (LIBs) are featured by higher energy density, higher cell voltage, less memory effect, very good cycle life, light weight, environmental friendliness and thus have found wide application in the area of communication and portable instruments. LIBs are also regarded as the most promising candidate for powering the next generation of hybrid electric vehicles (HEVs) and plug-in hybrids (PHEVs) [1,2].

Recently, heat-sealable multi-layer aluminum foil has been used in the Li-ion pouch cell design [3]. The electrical contacts in the pouch cell consist of conductive foil tabs that are welded to the electrode and sealed to the pouch material. Compared with conventional metallic LIBs (either cylindrical type or prism type), the pouch LIBs based on aluminum-composite-film provide higher flexibility in punching, lighter weight, better heat dissipation, lower price and make the most efficient use of space. The most suitable application for the pouch LIBs is cell phone and portable consumer electronics requiring ultra-thin enclosures.

To meet the high performance requirements of LIB packaging, the composite films must provide high barrier levels to gas, light, water, high electrolyte corrosion resistance as well as high mechanical and heat seal properties during the service life of LIBs. Therefore, in this tri-layer composite film, a polyamide (PA) layer was used as the outer layer to provide mechanical durability; an aluminum (Al) layer was used to block gas, light and water vapor, and the inner layer was selected as a polypropylene (PP) layer that contributes to electrolyte resistance and heat seal properties.

Many studies concern the tensile and fracture behavior of polymer-metal bi-layer composite films [4,5,6,7,8,9,10,11,12,13,14]. The deformation of the polymer layer during stretching was found to be very important for the tensile properties of the laminate. E. Andreasson et al. [14] studied micro-mechanisms of fracture in a laminate composed of an aluminum foil and a polymer film and found that the fracture or failure process of the freestanding aluminum is a localised plastic deformation and thinning until the cross section vanishes. In the freestanding Low-density polyethylene (LDPE), localised plastic deformation was not observed. Instead, plastic deformation occurs in diffuse regions that surround the crack tip. The plastic region increases to incorporate most of the test specimens at larger loads. In the laminate, the aluminum layer behaves similar as a freestanding layer, but the LDPE layer switched to localized deformation, seemingly forced to do so to compile with the deformation of the aluminum. On the other hand, the adhesion of the metal/polymer interface was also important in the deformation behavior of the laminates. Z. Suo et al. [4,6,8] suggested that when a laminate of a thin metal film on a tough polymer substrate is stretched, two processes—debonding and necking—take place and facilitate each other, leading to the rupture of the film. The metal film may rupture at strains ranging from a few percent to a few tens of percent, depending on the adhesion of the metal/polymer interface. E. Planes [7] reported the heat sealing properties of multilayers composed of one polyethylene layer and one or three polyethylene terephthalate layers coated with aluminum, and a comparison between the sole sealant film and multilayers was performed in terms of the range of optimal heat sealing parameters and mechanical behavior of seals.

However, in the case of a polymer-metal-polymer tri-layer composite film, the involved tensile and deformation behavior was more complicated, which has never been studied or reported before.

On the other hand, the heat seal behavior of polymer sealant was also investigated [15,16,17,18,19,20,21]. P. Meka et al. [15,16,17] quantitatively studied the effect of heat-seal variables (seal bar temperature, dwell time, and pressure) on the heat seal properties (seal strength, seal elongation, and seal energy) of PE films. Moreover, the roles of melting distribution and corona discharge treatment were also deeply investigated. Funasaka et al. [21] investigated the adhesive ability and solvent solubility of propylene-butene copolymer modified with maleic anhydride, which is used between oriented PP film and aluminum substrates, and observed correspondence between the amount of polar component and the T-peel strength and heat seal strength. K. Chaffin et al. [22] studied the welding behavior of PE/iPP laminates and proposed an interfacial polymer entanglement mechanism for the intrinsic properties of the laminates. T. Tervoort et al. [23] studied the effect of crystallization on the welding behavior of semicrystalline polymers by means of T-peel testing, using Ultra-high molecular weight polyethylene (UHMWPE) as a model polymer, and found that the cocrystallization across the interfaces is extremely efficient in enhancing the adhesive fracture energy. A. Hiltner, E. Baer et al. [20] investigated the adhesion of some propylene-ethylene copolymer to PP and high density polyethylene (HDPE), to compare them with other olefin copolymers such as compatibilizers for PP/HDPE blends. N. Mazzola et al. [19] studied the thermal behavior of sealant and heat seal properties of multilayer polyolefin films and found a linear correlation between heat seal initiation temperature and the temperature at with 40% of the polymer melted. A. Simanke [18] studied the microstructure and seal properties of four commercial linear low density polyethylenes (LLDPEs) and proposed that the comonomer distribution is one of the main factors that influence the seal properties. 

However, the performance of composite polymer-metal films has not been thoroughly studied. The studies above focused on the heat-seal properties of multilayer films, but the presence and influence of the metal layer have not been discussed. More recently, Z. Guo et al. [3] studied the heat seal properties of five different polymer-aluminum-polymer composite films. The effects of heat seal temperature and dwell time on heat seal strength and failure mode were studied.

In summary, to meet the high performance demands for LIB packaging, the tensile behavior and the roles of each layer during the tensile process should be investigated in detail. Furthermore, the roles of each layer in the heat seal properties of the composite film are also of great importance. However, as far as we are concerned, these important points are not deeply understood.

In the present study, the tensile performances and fracture behaviors of the individual layers of PA, Al and PP, as well as the laminated films of PA-Al, PP-Al and PA-Al-PP, were investigated to explore the contributions of each layer to the tensile behavior of the PA-Al-PP composite films for pouch LIBs. Moreover, two PP layers with different orientation status were used to prepare the laminated films, so as to comparatively study the role of PP orientation in the peeling and tensile behavior of heat seal strength (*HSS*) of the laminated film. The related mechanism was proposed.

## 2. Experimental Section

### 2.1. Materials

iPP, tradename T38F (Lanzhou OilChem Corp., Lanzhou, China) with average isotacticity 97.6%, weight molecular weight 347,200, polydispersity index = 3.63, was used. Detailed structure characterizations can be found in previous studies [24,25].

Polyolefin adhesive: a mixture of LOTADER 4210 (ARKEMA Innovative Chemistry, France) and Versify 3200 (Dow Chemistry Company, Midland, MI, USA) was used.

Biaxially oriented polyamide film (BOPA film), tradename BOPA-15U, produced by simultaneous biaxially orientated technology, was provided by Cangzhou Mingzhu Plastic Co., Ltd., Cangzhou, China.

Aluminum foil (Al foil), flexible package grade, was purchased from Zhejiang Dingsheng Aluminum Corporation, Ltd., Hangzhou, China.

Two-component polyurethane adhesive (PU adhesive), tradename LOCTITE 8103, was purchased from Henkel Corp., Düsseldorf, Germany.

To benefit the discussion, the polymer-metal-polymer tri-layer laminated films used in this study were shortened to PCPF.

It should be noted that the adhesive between the layers played an important role in the tensile, peeling and even the heat seal behaviors. If alternative adhesives were used, the results and summaries may be totally different. Therefore, the selection of adhesives is carefully considered according to the commercial composite films for LIBs.

### 2.2. Sample Preparation

**Scheme 1:**
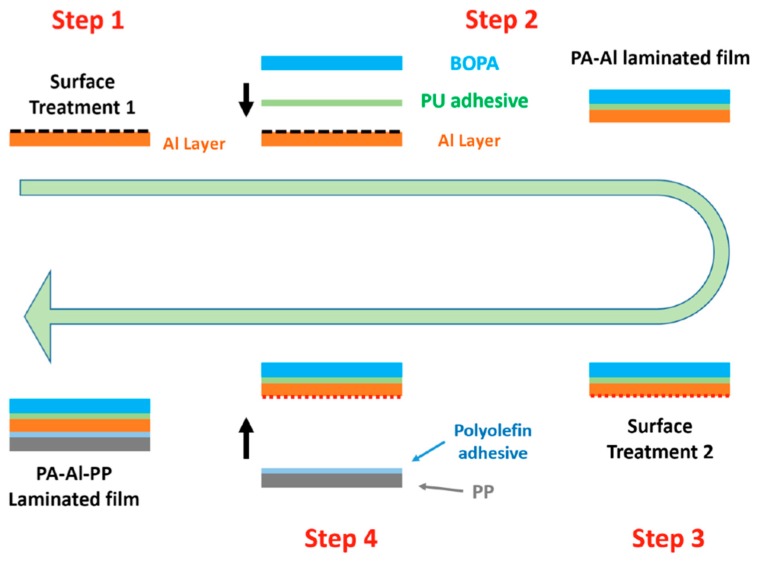
Schematic illustration of the process to prepare PA/PU adhesive/Al foil/Polyolefin adhesive/PP laminated film.

**Scheme 2:**
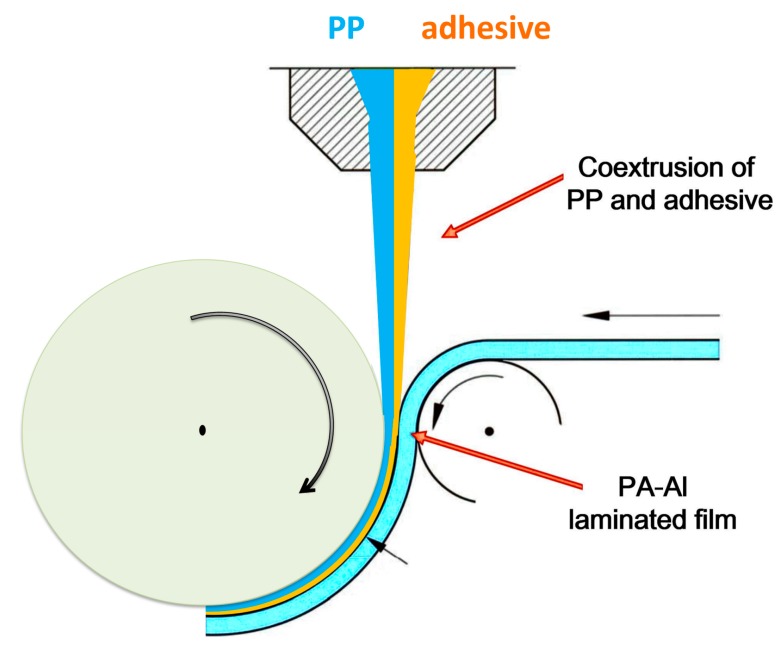
Schematic illustration of the extrusion coating process to prepare the PA-Al-PP laminated film.

The laminated film was prepared according to industrialized manufacturing procedures as follows (Scheme 1 and Scheme 2):

**Step one**, the surfaces of aluminum foil were washed, and then one surface was subjected to corona surface treatment to enhance its adhesion to adhesives;

**Step two**, the BOPA film, PU adhesive and the surface-treated Al foil were laminated by dry lamination process using a Dry Compounded Machine to obtain the PA-Al laminated film. Then, a thermal aging process was applied to enhance the adhesion strength of the PU adhesive;

**Step three**, chemical surface treatment was applied on the other side of the Al foil surface of the PA-Al laminated film to enhance its resistance to the electrolyte and solvent;

**Step four**, the laminated film of PP-PA-Al was prepared by an extrusion coating process, as can be seen n Scheme 2. The PP and adhesive were first coextruded via the extruder die and then laminated with the PA-Al film using a set of rolls. Finally, an online annealing process was applied.

### 2.3. Characterizations

#### 2.3.1. Scanning Electron Microscope (SEM)

The morphology observation (Scanning Electronic Microscopy, SEM, JEOL, Tokyo, Japan) was performed on a JSM-5900 LV environmental scanning electron microscope at an accelerating voltage of 20 kV. Before SEM characterizations, the fractured surfaces of all the samples were coated with a thin layer of gold by ion sputtering [26].

#### 2.3.2. Fourier Transform Infrared Spectroscopy (FT-IR)

Fourier transform infrared spectroscopy (FTIR) (Madison, WI, USA) was performed to detect the orientation of the amorphous and crystalline phases. This method is based on the absorption of infrared light at certain frequencies corresponding to the vibration modes of atomic groups present within the molecule. A Nicolet 560 FTIR instrument from Thermo Electron was used for FTIR measurements, and the beam was polarized in two orthogonal directions, parallel and perpendicular to a reference axis, by a zinc selenide wire grid polarizer. These two absorption values should be different, and their ratio is defined as the dichroic ratio, *D*.

The Herman orientation function of this vibration was obtained according to the method of Tabatabaei et al. [27,28]:

(1) $$f_{i,MD}=-\frac{D-1}{D+2}$$

where *D* is the ratio of the absorption parallel and absorption perpendicular to the machine direction. For polypropylene, absorption at the wave number of 998 cm^−1^ is attributed to the crystalline phase (*c* axis) whereas absorption at the wave number of 972 cm^−1^ is due to the contribution of both crystalline and amorphous phases. The orientation of crystalline phase (*f_c_*) and average orientation function (*f_av_*) could be calculated by Equation (1). Moreover, since the 2723 cm^−1^ band is the only amorphous band of PP, the orientation of the amorphous phase (*f_am_*) could be calculated by Equation (2). The transition moment angle at 2723 cm^−1^ is 90° [28].

(2) $$f_{am}=\frac{2(D_{2723}-1)}{D_{2723}+2}$$

#### 2.3.3. Differential Scanning Calorimeter (DSC)

All the calorimetric experiments were performed with a Mettler Toledo DSC1 differential scanning calorimeter (DSC) (Mettler-Toledo, Zurich, Switzerland), under a nitrogen atmosphere (50 mL/min). The temperature scale calibration was performed using indium as a standard to ensure reliability of the data obtained. In order to ensure the homogeneity of samples and the good contact between sample and pan, the virgin polymer was molded at 190 °C, 10 MPa for 5 min into sheets of uniform thickness about 500 μm. Then, 5 mg round samples were punched out of the sheets.

The degree of crystallinity (*X_c_*) of the samples was calculated by the following equation [29,30]:

(3) $$X_{c}=\frac{\Delta H_{m}}{\Delta H_{m}^{0}}$$

where ∆*H_m_* is the DSC measured value of fusion enthalpy, and ∆*H*^0^*_m_* is the fusion enthalpy of the completely crystalline iPP. The values of ∆*H*^0^*_m_* for PP and PA were selected as 209 J/g and 255 J/g, respectively.

#### 2.3.4. Mechanical Properties Measurement

**Tensile behavior:** Rectangular strips with a width of 15 mm were carefully cut from the films along the machine direction (MD) and transverse direction (TD). The tensile testing was conducted on a universal tensile testing machine (Instron 4302, Instron Corporation, Turin, Italy) with a load range of 0–1000 N. The gauge length was 50 mm. All the tensile tests were performed at 25 °C with an elongation rate of 50 mm/min.

**T-Peel test:** The measurement of heat seal strength (*HSS*) was performed by tension peel testing on universal tensile testing machine (Instron 4302, Instron Corporation, Turin, Italy) [3]. The specimens were aligned with seal lines perpendicular to the direction of tension. All tests were conducted at about 25 °C with a strain rate of 50 mm/min. During the test, the maximum load was recorded. The *HSS* was defined as the maximum tensile load divided by the sample width, in units of N/15 mm. The *HSS* was averaged over five samples for every heat-seal condition. In addition, the failure mode of each test was carefully examined and investigated in the study.

## 3. Results and Discussion

### 3.1. Overview

#### 3.1.1. Lamination Structure Observation

**Figure 1:**
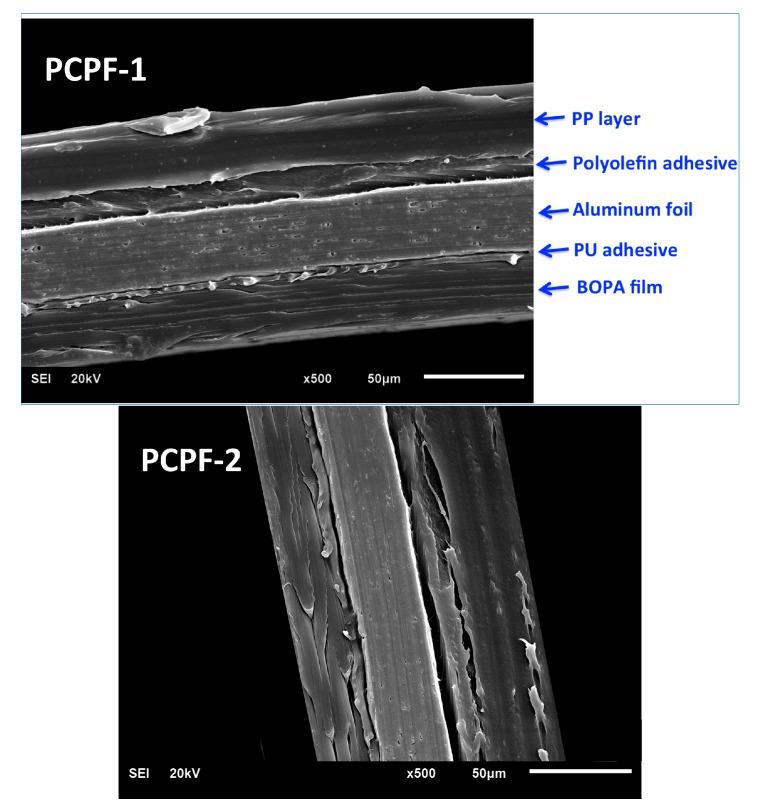
SEM images of the fractured surface of PCPF-1 and PCPF-2.

The SEM images of the cross-section of the composite film (PCPF-1 and PCPF-2) are shown in Figure 1. The five-layered structure, including three main layers and two adhesive layers, can be clearly observed. The affiliations of these layers are also added in Figure 1.

Figure 1 clearly reveals the five-layer structure in the composite film, where the cross-section morphology of PCPF-1 and PCPF-2 is quite similar. The total thickness of the composite film is about 125 μm. The middle layer is aluminum foil (Al layer) with a thickness of about 40 μm, while on its two sides, the PP layer and PA layer with a thickness of about 40 μm and 30 μm, respectively, can be observed. Between the PP layer and Al foil, there is polyolefin adhesive (thickness 15 μm), which provides adhesion between PP/Al, while on the other hand, there is polyurethane adhesive (thickness 3–5 μm) between the PA layer and Al layer.

These different layers contribute to different properties, which together enable the composite film to meet the critical performance requirement for pouch LIBs: The PP layer provides heat-seal properties, certain resistance of electrolyte and mechanical properties, while the PA layer contributes its superior mechanical properties. The Al layer in the middle gives the laminated film superior gas and water barrier properties. In this way, the easily sealable laminated film with high barrier property and superior mechanical properties for pouched LIB application is obtained. 

#### 3.1.2. DSC Analysis

**Figure 2:**
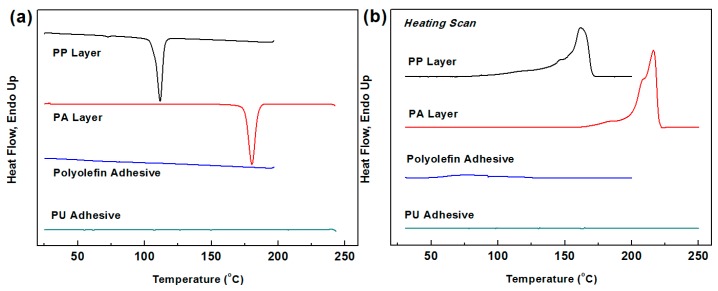
DSC (**a**) cooling and (**b**) subsequent heating curves of the materials involved in the composite film.

The DSC cooling curves after elimination of thermal history, as well as the subsequent melting curves of each layer are plotted as shown in Figure 2. The obtained crystallization and melting parameters are listed in Table 1.

As can be seen from Figure 2 and Table 1, the crystallization peak temperatures *T_c_* of the PP layer and PA layer after elimination of the thermal history are 110.2 °C and 180.5 °C, respectively. For the polyolefin adhesive, its crystallization peak can hardly be observed, indicating that its isotacticity is very low [24,30]. No crystallization peak can be observed for the PU adhesive, since it is already crosslinked and cannot crystallize.

From the aspect of melting, the melting points *T_m_* and degrees of crystallinity *X_c_* of PP and PA are 159.3 °C and 216.2 °C, 34.2% and 36.1%, respectively. The *T_m_* and *X_c_* of polyolefin adhesive are only 85.4 °C and 7.8%, respectively, reflecting the very poor crystallizability of the polyolefin elastomer [31,32]. Moreover, no melting peak of the PU adhesive can be found, indicating that it cannot crystallize.

#### 3.1.3. Tensile Properties of the Laminated Films

Although the gas and water barrier properties of aluminum foil are extremely high, suffering from its poor ductility, permanent deformation and even fracture will easily take place when the aluminum foil is under even slight scraping or stretching [3]. Therefore, it must be laminated with polymer films to enhance its ductility and to give it the heat seal property.

To deeply investigate the relationship between the multi-layer structure and final properties of the composite film and to clarify the contribution of each layer to the mechanical properties of the composite film, in this section, we carefully prepared the composite films with different constitutions, including PP/Al, PA/Al and PA/Al/PP. Their tensile behaviors are investigated.

**Figure 3:**
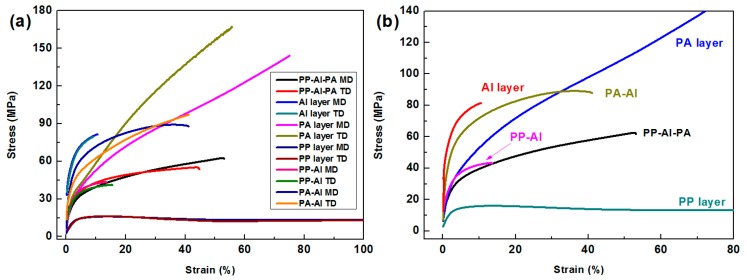
Stress-strain curves of composite films (**a**) results of two directions, machine direction (MD) and transverse direction (TD); (**b**) measurement from MD.

Figure 3a represents the stress-strain curves of PCPF-1 at both MD (machine direction) and TD (transverse direction), and Figure 3b shows the stress-strain curves of samples in the MD direction, including single-layer PP, Al, PA films, and the composite films of PA-Al, PP-Al, PP-Al-PA. From Figure 3a, the tensile strength and elongation at the breakage points of the specimens are shown in Table 2.

Figure 3 and Table 1 reveal that for the single-layer films, PA exhibits the highest tensile strength of more than 144 MPa, and its elongation at breakage lies between 50–70%; PP has the lowest tensile strength of about 23 MPa; however, it exhibits the highest elongation at a breakage of more than 600%; Al foil has high tensile strength of about 80 MPa, but its elongation at breakage is as low as 9–11%.

After lamination, elongation at breakage of PA-Al is evidently enhanced compared with single Al foil, indicating that using PU adhesive, PA and Al foil are closely bonded, and the poor ductility of Al foil is evidently reinforced. Meanwhile, its tensile strength is also increased, indicating that the tensile stress is released from Al to PA. With respect to the PP-Al composite film, its tensile strength and elongation at breakage are very low compared with PA-Al, which might be attributed to the very low tensile strength of PP. When the tensile stress is applied, the “soft” PP layer cannot efficiently share the tensile stress in Al layer; therefore, the deformation and rupture of each layer mainly occur in their own way, and the final tensile property of PP-Al can hardly be significantly enhanced.

For the PA-Al-PP laminated film, its tensile strength and elongation at break are higher compared with PA-Al, indicating that the addition of the PP layer can further reinforce the tensile properties of the laminated film.

On the other hand, considering the tensile results in MD and TD, only slight differences in their tensile properties can be seen.

**Figure 4:**
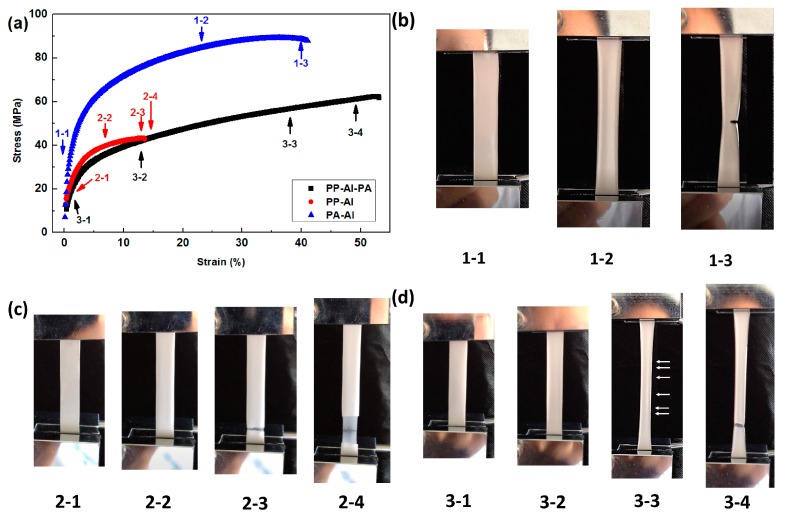
(**a**) Stress-strain curves and morphological evolution of composite films during stretching (**b**) PA-Al; (**c**) PP-Al and (**d**) PA-Al-PP laminated film.

To further explore the contributions of the PP layer and PA layer to the PA-Al-PP laminated film, the morphology evolution of PP-Al, PA-Al and PA-Al-PP during the tensile process are further studied. The results are shown in Figure 4. 

As can be seen from Figure 4, the deformation and rupture processes of these three types of laminated films are quite different. For PA-Al, when the tensile stress is applied, the PA layer and Al layer deform simultaneously at the beginning, and the width of the specimen decreases gradually, indicating that plastic deformation takes place. As the strain increases, the tensile stress concentrates, which cannot be efficiently dispersed, and the ruptures of the PA layer and Al layer take place simultaneously in the boundaries. The elongation at breakage and the tensile strength of the PA-Al film are 41.2% and 87.5 MPa, respectively, both higher compared with the single Al layer, which can be explained as follows: PA has higher tensile strength compared with Al, which can share a large amount of stress from Al during stretching, resulting in the coordination deformation of PA and Al with the characteristic of plastic deformation. 

On the other hand, for PP-Al, when tensile stress is applied, the width of the specimen remains almost unchanged before the rupture of the Al layer. When the strain increases to more than 10%, the Al foil is broken in a brittle rupture manner, while the elongation and plastic deformation of PP layer continue.

For the PA-Al-PP composite film, it can be observed that during the tensile process, the characteristic of plastic deformation is observed, and all the layers deform simultaneously in the same manner; the decrease of width and necking can be seen as the strain increases. At higher strain, many tiny cracks can be seen from the boundary of the Al layer, as indicated by arrows in Figure 4d. Surprisingly, with the help of the PA and PP layers, the further enlargement of these cracks are restrained, resulting in higher tensile tolerance of the laminated films. Finally, as the strain further increases, the sample is fractured, exhibiting high elongation at a breakage of 53.2% and a high tensile strength of 61.5 MPa.

**Figure 5:**
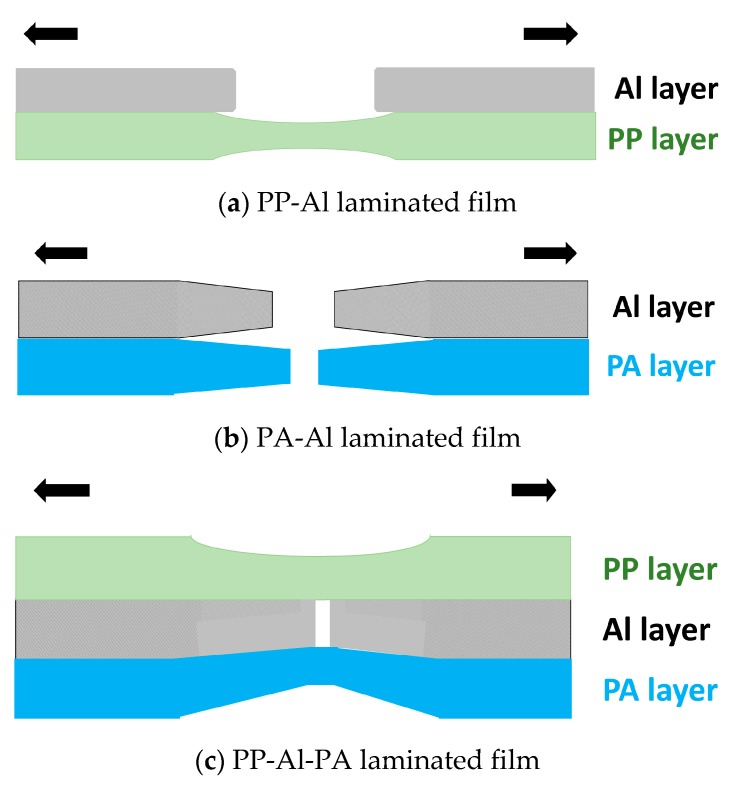
Schematic illustration of the roles of the PA and PP layers in the final tensile behavior of the PA-Al-PP composite film. (**a**) PP-Al; (**b**) PA-Al; (**c**) PP-Al-PA. The arrows indicate the tensile direction.

Based on the experimental results above, we proposed the following mechanism describing the roles of the PA and PP layers in the final tensile behavior of the PA-Al-PP composite film, as shown in Figure 5.

As is illustrated in Figure 5, for PP-Al, since the tensile strength of the PP layer is too low compared with Al, PP cannot efficiently share tensile stress from Al and cannot reinforce the ductility of Al. During stretching, PP and Al are broken individually in their own manner.

For PA-Al, owing to the high tensile strength and plastic film nature of the PA layer, it can share a large amount of stress from the Al layer, helping the Al layer to deform in the plastic deformation manner. However, when cracks appear on the Al layer, PA can hardly restrain the further enlargement of these cracks. The Al layer and PA layer break together.

For the PA-Al-PP laminated film, it can be seen that not only the tensile strength can be efficiently shared from Al layer, but also the small cracks can be restrained with the help of the PP layer (compared with Figure 5b). In this way, the laminated PA-Al-PP film with balanced tensile strength and elongation at break is obtained.

In summary, in the PA-Al-PP laminated film, PA with high tensile strength can release a large amount of tensile stress from the Al layer during stretching, while the PP layer can restrain the further development of the small cracks on the boundary of the Al layer, preventing the final rupture of the laminate. It should also be noted that the adhesives between the PP-Al and PA-Al layers also play a determining role in the final properties of the laminated film. Tight and even bonding between each layer is a prerequisite and the foundation for obtaining the laminated PA-Al-PP with desired tensile properties [4,5].

### 3.2. Fourier Transform Infrared Spectroscopy Analysis on PP Layer

To explore the role of PP orientation in the final properties of the laminated film, we have prepared two PP layers with different orientation status, and thus two laminated films, PCPF-1 and PCPF-2. The FT-IR spectrums of the PP layers of PCPF-1 and PCPF-2 in two directions are shown in Figure 3. The calculation results of the orientation factors of crystalline phase, amorphous phase and average are listed in Table 3.

**Figure 6:**
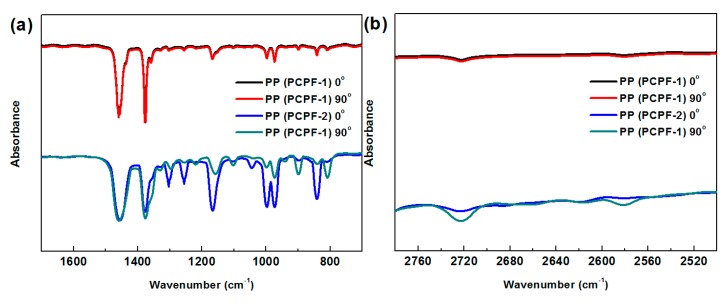
Spectrum of Fourier Transform Infrared Spectroscopy of the PP layers of PCPF-1 and PCPF-2 at different ranges. (**a**) FT-IR spectrums in whole range; (**b**) FT-IR spectrums in the range of 2500–2800 cm^−1^.

Figure 6 and Table 3 reveal that the IR spectrum of the PP layer of PCPF-1 at two different directions is almost the same, and its orientation factors are zero; on the other hand, for the PP layer of PCPF-2, it can be seen that its *f_c_*, *f_am_* and *f_av_* are 0.294, 0.350 and 0.309, respectively, indicating that although a thermosetting process is performed, the PP layer of PCPF-2 retains a certain degree of orientation along the machine direction.

### 3.3. Peeling Behavior of PCPF-1 and PCPF-2

**Figure 7:**
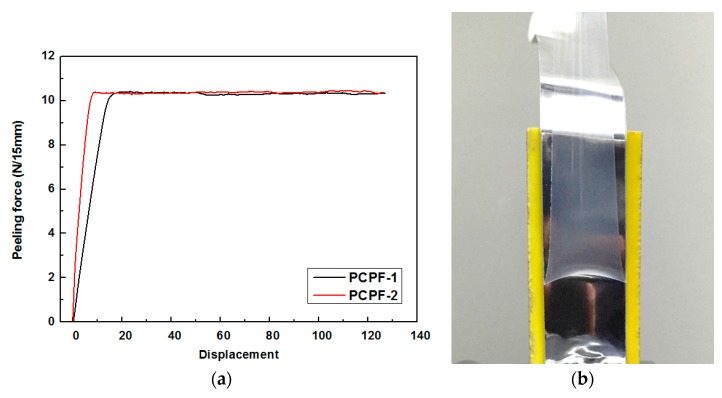
(**a**) Peeling curves of PP-Al of PCPF-1 and PCPF-2 (**b**) image of PCPF-1 during the peeling test.

The peeling test is performed on the PP-Al layer of PCPF-1 and PCPF-2 to explore the impact of PP orientation in this process. The obtained peeling curves and morphology image are shown in Figure 7.

Figure 7 reveals that PCPF-1 and PCPF-2 exhibit almost the same peeling force, about 10.3 N/15 mm, indicating that the peeling force is mainly determined by the situation of bonding, not the orientation of the PP layer. When the samples are bonded under the same condition, the final peeling forces are quite similar, too. Interestingly, at a small displacement of less than 20 mm, it can be seen that the slopes of the peeling curves of PCPF-1 and PCPF-2 are quite different; the slope of PCPF-2 is obviously higher than that of PCPF-1.

On the other hand, Figure 7b reveals that after the PP layer is peeled from the Al layer, a smooth surface of the Al layer without PO adhesive can be observed. During the peeling process, the plastic deformation of the PP layer and the separation between the PO adhesive and Al layer take place simultaneously. Meanwhile, the peeling frontier is not linear, indicating that the stress applied on the frontier is not uniformly distributed.

### 3.4. Heat Seal Properties

Since the heat seal properties of the laminated film are directly related to its PP layer, the orientation status of the PP layer might play an important role in the process. Therefore, in this section, the heat seal properties of PCPF-1 and PCPF-2 are investigated, and the roles of heat seal temperature (*T_H_*) and dwell time in the heat seal strength (*HSS*) and failure behavior are studied.

- *Effects of heat seal temperature*

**Figure 8:**
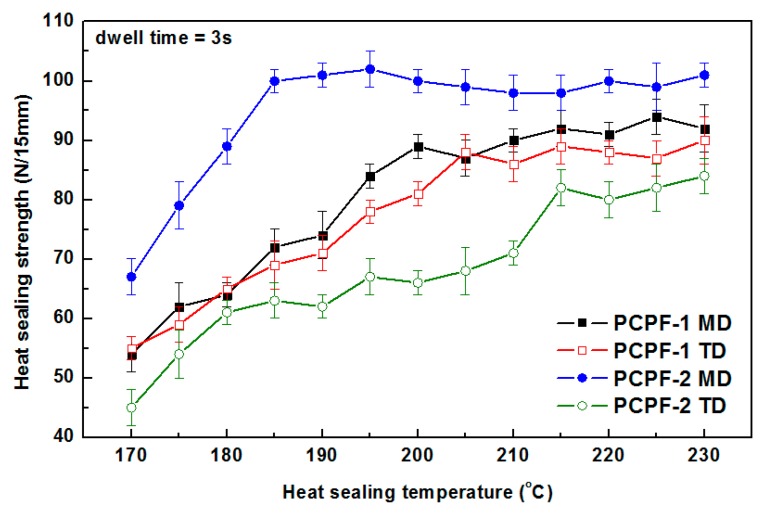
Heat seal strength (*HSS*) of samples as a function of heat temperature at dwell time of 3 s.

The experiment is performed at the heat seal temperature (*T_H_*) of 170–230 °C, dwell time of 3 s and heat seal pressure of 1 MPa. The obtained heat seal strength (*HSS*) is plotted as a function of *T_H_* as shown in Figure 8.

As can be seen from Figure 8, for both PCPF-1 and PCPF-2, the *HSS* increases gradually with the increase of *T_H_*. Meanwhile, a critical transition heat seal temperature *T_crit_* can be observed: when the heat seal temperature is higher than *T_crit_*, the *HSS* reaches a plateau region and does not increase evidently with the further increase of *T_H_*.

Comparing the results of PCPF-1 and PCPF-2, it can be seen that the *HSS* values in the MD and TD directions are quite similar. At the same *T_H_*, the *HSS* of PCPF-1 in MD direction is slightly higher than that in TD, and the corresponding *T_crit_* in MD and TD directions is 195 °C and 200 °C, respectively. However, for PCPF-2, the *HSS* in MD direction is obviously higher than its counterpart in the TD direction in all the heat seal temperature ranges studied, and its *T_crit_* in the MD and TD directions is 185 °C and 215 °C respectively. Moreover, the *HSS* values of the samples above the plateau region are also quite different. Generally, from the highest to lowest, the ranking is PCPF-2 MD > PCPF-1 MD > PCPF-1 TD > PCPF-2 TD.

The results above indicate that unlike PCPF-1, the heat seal properties of PCPF-2 in the MD and TD directions are quite different from each other, which should be related to its orientation in the PP layer.

**Figure 9:**
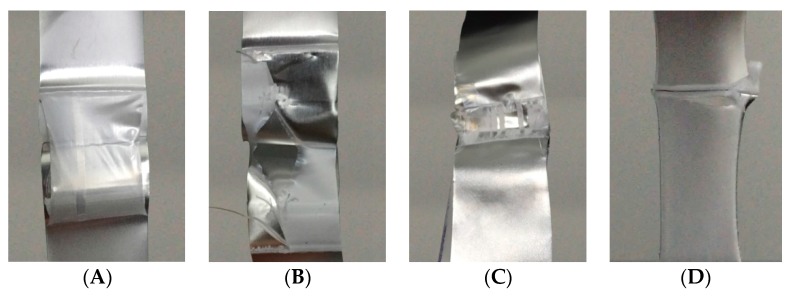
Four typical failure types of the heat-sealed specimens during stretching. (**A**) Type A; (**B**) Type B; (**C**) Type C and (**D**) Type D.

To understand fully, the failure behavior of the heat sealed samples during tensile stretching is also determined. Generally, the failure behavior can be summarized into four failure types (Types A–D) as shown in Figure 9:

Type A: When the tensile stress is applied, the heat seal frontier remains unchanged. Meanwhile, the adhesion between PP layer and Al foil is broken. With the further increase of the strain, only the plastic deformation of freestanding PP layer takes place;

Type B: The failure first takes place in the heat seal frontier, which is split into two pieces. With a further increase in the tensile stress, the separation between the PP layer and Al layer, as well as the failure of PP layer take place at the same time;

Type C: Similar to Type B, the failure first takes place in the heat sealed frontier. Interestingly, with a further increase n the tensile stress, the PP layer is split into many tiny pieces, accompanied with the separation between PP and Al;

Type D: At the beginning of the tensile stress, no evident failure is observed. Instead, evident necking in the laminate film near the heat seal frontier takes place (as indicated by arrows in Figure 9D), and the width of the laminate film decreases gradually. Meanwhile, the width of the heat seal frontier remains unchanged. With the increase of tensile stress, the strain of the laminate film along the tensile direction increases evidently, and finally, failure takes place in the heat seal frontier or in the laminate film nearby. This type of failure usually happens when *HSS* reaches 90 MPa or higher, reflecting that all the layers are tightly laminated, and the two PP layers are perfectly sealed. 

As can be clearly seen from Figure 9 and Table 4, the failure behavior of the specimen is closely related to *HSS*. Generally, when *HSS* ≤ 70 MPa, mainly the failure mode of Type A occurs, supplemented with Type B; when 70 MPa ≤ *HSS* ≤ 90 MPa, the mixed failure types of Type B and Type C take place; for *HSS* higher than 90 MPa, only the failure mode of Type D can be observed. In summary, it is found that the failure type is an indicator of the *HSS* of the sample. In fact, the *HSS* value can be far higher than the tensile strength of the PP layer, which can be even higher than the tensile strength of the laminate film, only if each layer is tightly laminated and shares the tensile stress efficiently.

- *Effects of dwell time*

**Figure 10:**
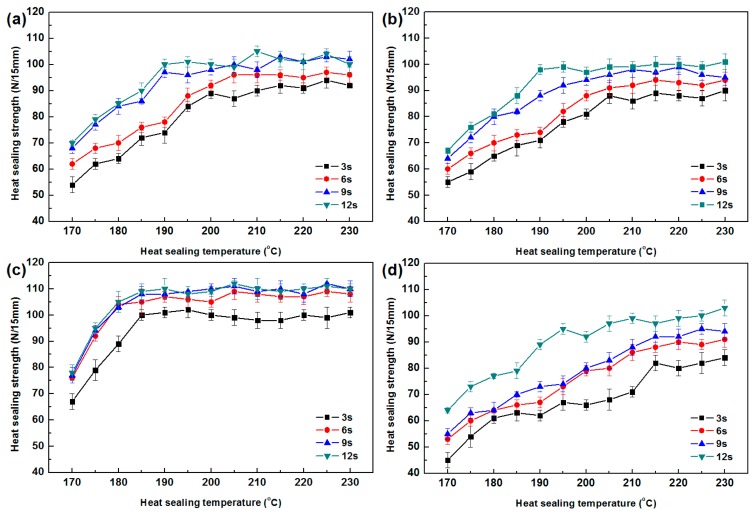
Heat seal strength (*HSS*) of the samples as a function of the heat temperature at various dwell times. (**a**) PCPF-1 (MD); (**b**) PCPF-1 (TD); (**c**) PCPF-2 (MD) and (**d**) PCPF-2 (TD).

The *HSS* values of samples heat sealed at different dwell times are measured and plotted as a function of heat seal temperature as shown in Figure 10. To benefit comparison, the *Y*-axis scales of Figure 10a–d are the same.

Figure 10 suggests that for all the samples, at given *T_H_*, the *HSS* value increases with the increase in dwell time, and the critical transition heat seal temperature *T_crit_* decreases gradually.

However, the increase margin of *HSS* and decrease rate of *T_crit_* of the samples are quite different from each other. For PCPF-2 MD, when the dwell time is 6 s, the *HSS* value will not increase significantly with a further increase in dwell time. For PCPF-1 MD, 9 s and higher dwell time has a similar effect on the *HSS* value; for PCPF-1 TD and PCPF-2 TD, the *HSS* value increases continuously with the increase in dwell time. Moreover, when the dwell time is 12 s, the *HSS* values of the samples can be ordered from high to low as: PCPF-2 MD > PCPF-1 MD > PCPF-1 TD > PCPF-2 TD, indicating that given sufficient dwell time and heat seal temperature, the *HSS* value that the samples can achieve is still related to its orientation status of the PP layer.

On the other hand, for PCPF-1 MD and PCPF-1 TD, the critical transition heat seal temperature (*T_crit_*) decreases gradually from around 205 °C to 190 °C with an increase in dwell time from 3 s to 12 s, while for PCPF-2 MD and TD, the *T_crit_* decreases from 185 °C and 210 °C to 180 °C and 205 °C, respectively, indicating that the variation of *T_crit_* of the specimens is also closely related to its orientation in the PP layer.

With respect to the failure behavior, an evident relationship between *HSS* and failure type is clearly observed again in this section, which is in accordance with the observation above.

In summary, the important heat seal properties of the laminated film are closely related to the orientation status of the PP layer. The qualitative variation of the heat seal properties with the increase of orientation degree in the MD direction is listed in Table 5.

Since the PP layer is directly related to the heat seal properties of the laminate film, its orientation status is a determining factor of the heat seal properties, i.e., the *HSS*, the dwell time needed to obtain the desired *HSS*, the critical transition heat seal temperature (*T_crit_*), the highest *HSS* value on the platform and the difference of *HSS* between MD and TD.

A higher orientation degree of the PP layer in the MD direction leads to a higher difference in *HSS* between the MD and TD direction. With the increase of the orientation degree in MD, the *HSS* increases, the time needed to obtain the desired *HSS* decreases, and both the critical transition heat seal temperature (*T_crit_*) and the highest *HSS* value on the platform increase; while in TD, as the orientation degree in MD increases, the *HSS* decreases, the time needed to obtain the desired *HSS* increases, and both the critical transition heat seal temperature (*T_crit_*) and the highest *HSS* value on the platform decrease.

## 4. Conclusions

In this study, the tensile performances and fracture behaviors of the single layers of PA, Al and PP, as well as the laminated films of PA-Al, PP-Al and PA-Al-PP, were investigated to explore the contributions of each layer to the tensile behavior of the PA-Al-PP composite films for pouch lithium-ion batteries. The results revealed that in the PA-Al-PP composite film, the PA layer with the highest tensile strength can release a large amount of tensile stress from the Al layer during stretching, while the PP layer with the lowest tensile strength can restrain the further development of the small cracks on the boundary of the Al layer under tensile stress; in this way, the high tensile strength and plastic deformation of the composite film are obtained.

Moreover, two PP layers with different orientation status were used to prepare the laminated films to explore the role of PP orientation in the peeling and tensile behavior of heat seal strength (*HSS*) of the laminated film. The results revealed that the orientation status of the PP layer has little influence on the peeling force of the sample; however, it influences the slope of the peeling curve. Both the orientation status and the heat seal conditions were important factors in determining the *HSS* and failure behavior of the sample. A higher heat seal temperature (*T_H_*) leads to higher *HSS* and less dwell time to achieve the relatively high *HSS*, while with under an increase in dwell time, *HSS* increases gradually and the onset heat seal temperature *T_crit_* (above which the plateau region of the *HSS* value is reached) decreases. Four failure types of the heat sealed samples were observed, and a clear correspondence between *HSS* and failure types was found. From the aspect of the PP layer orientation, it was found that in the case of the PP layer without orientation, its *HSS* in MD and TD are quite similar; while in the case of PP with orientation, a significant difference in heat seal behavior in MD and TD was found: in MD, the composite film exhibit higher *HSS*, lower *T_crit_* and less time to achieve high *HSS*, while in TD, lower *HSS*, higher *T_crit_* and more time to obtain high *HSS* were observed; the related mechanism was proposed based on the oriented molecules in the PP layer. In general, the PP orientation status was very important in the heat seal behavior of the composite film. The results in this study also elucidated that for the PP-Al-PA composite film, only if the tensile stress was released by each layer efficiently during the *HSS* measurement could the composite film exhibit the desired high *HSS* that was even higher than its tensile strength. Therefore, adequate lamination of each layer is very important for the final properties of the film.

## Figures and Tables

**Table 1:** Crystallization and melting parameters of the materials involved in composite film.

Sample	Cooling Process			Subsequent Heating Process	
	*T_c_* (°C)	*T_conset_* (°C)	*T_cendset_* (°C)	*T_m_* (°C)	*X_c_* (%)
**PP Layer**	110.2	116.1	103.0	159.3	34.2
**PA Layer**	180.5	187.4	174.0	216.2	36.1
**Polyolefin adhesive**	-	-	-	85.4	7.8
**PU adhesive**	-	-	-	-	-

**Table 2:** Parameters of the tensile behavior of the samples.

Samples	Tensile Strength (MPa)		Elongation at Break (%)	
	MD	TD	MD	TD
**PP Layer**	25.3 ± 0.1	23.4 ± 0.1	596.5 ± 24.1	604.7 ± 32.0
**PA Layer**	144.5 ± 0.4	167.8 ± 0.3	75.3 ± 5.2	55.5 ± 3.1
**Al Layer**	81.6 ± 0.2	80.5 ± 0.1	10.8 ± 1.1	9.4 ± 0.7
**PP-Al**	43.3 ± 0.1	40.9 ± 0.1	13.7 ± 1.2	14.7 ± 1.1
**PA-Al**	87.5 ± 0.1	97.5 ± 0.2	41.2 ± 2.9	41.4 ± 2.2
**PP-Al-PA**	61.5 ± 0.1	55.9 ± 0.1	53.2 ± 3.1	47.5 ± 2.5

**Table 3:** Orientation functions of the crystalline phase (*f_c_*), average (*f_av_*) and the amorphous phase (*f_am_*) for the PP layers of PCPF-1 and PCPF-2.

Samples	*f_c_*	*f_av_*	*f_am_*
PP layer (PCPF-1)	0.000	0.000	0.000
PP layer (PCPF-2)	0.294	0.309	0.350

**Table 4:** Failure behavior of the heat-sealed specimens during stretching.

Heat Seal Temp	170 °C	180 °C	190 °C	200 °C	210 °C	220 °C	230 °C
PCPF-1 MD	AAAAB	AAAAB	BBBCC	BCCCD	BBCCD	BBDDD	DDDDD
PCPF-1 TD	AAAAB	AAAAB	BBBBC	BBCCC	BBCCD	BCDDD	DDDDD
PCPF-2 MD	AAABB	BBBCC	CDDDD	DDDDD	DDDDD	DDDDD	DDDDD
PCPF-2 TD	AAAAA	AAABB	AAAAB	AAABB	ABBBC	BBBCC	BBCCD

**Table 5:** Variation of the heat seal parameters with the increase of orientation degree in the MD direction of the PP layer.

Heat Seal Parameters	MD	TD
*HSS*	+	−
Dwell time needed to obtain desired *HSS*	−	+
Critical transition heat seal temperature (*T_crit_*)	−	+
Highest *HSS* value on the platform	+	−
Difference of *HSS* between MD and TD	+	
